# Features of Translocation of Copper Nanoparticles in *Mentha spicata* L. and Extraction into Infusion

**DOI:** 10.3390/plants14213318

**Published:** 2025-10-30

**Authors:** Alexandra Peshkova, Inga Zinicovscaia, Liliana Cepoi, Ludmila Rudi, Tatiana Chiriac, Serghei Corcimaru, Nikita Yushin, Rikus le Roux

**Affiliations:** 1 Joint Institute for Nuclear Research, 6 Joliot-Curie Str., 141980 Dubna, Russia; ynik_62@mail.ru; 2 Doctoral School of Natural Sciences, Moldova State University, M. Kogalniceanu Str., 75A, MD-2009 Chisinau, Moldova; 3 Horia Hulubei National Institute for R&D in Physics and Nuclear Engineering, 30 Reactorului Str., 077125 Măgurele, Romania; 4 Institute of Microbiology and Biotechnology, Technical University of Moldova, 1 Academiei Str., MD-2028 Chisinau, Moldova; liliana.cepoi@imb.utm.md (L.C.); ludmila.rudi@imb.utm.md (L.R.); tatiana.chiriac@imb.utm.md (T.C.); serghei.corcimaru@imb.utm.md (S.C.); 5 School for Science and Technology, Faculty of Military Science, Stellenbosch University, Saldanha 7395, South Africa; rikusr@sun.ac.za

**Keywords:** copper nanoparticles, foliar spraying, root irrigation, spearmint, biochemistry, soil microbial activity, risk assessment

## Abstract

Metal nanoparticles are increasingly used in industry and agriculture to enhance crop yields and combat plant diseases. Their widespread application has led to exposure across all ecosystem components, including humans. However, there is a lack of a comprehensive assessment of the effect of copper nanoparticles on spearmint plants, applied in different ways (root and foliar) in a wide range of concentrations. The effect of copper nanoparticles at the concentration range of 1–100 mg/L on *Mentha spicata* L. plants under root exposure and foliar spraying conditions was studied during a 28-day experiment. Copper content in soil and spearmint segments was determined using inductively coupled plasma atomic emission spectroscopy, while the morphology of nanoparticles was characterized using transmission electron microscopy. Foliar spraying showed an inverse relationship between the concentration of copper in solution and root uptake. The highest copper uptake in roots of 27.51 mg/kg was attained at a nanoparticle concentration of 1 mg/L. Root exposure inhibited soil microbial activity, with copper mainly accumulating in soil (up to 950.2 mg/kg) and roots (up to 150.5 mg/kg). Both application methods stimulated pigment production and antioxidant activity, with root irrigation showing a more pronounced effect. Copper extraction efficiency varied (3–64%) depending on exposure method and concentration, raising concerns about transfer along the food chain. Health risk assessment associated with consumption of herbal infusions, prepared from copper-contaminated spearmint leaves, did not show adverse effects when copper levels in the infusion remained below 1.53 mg/L.

## 1. Introduction

The industrial production and use of engineered copper nanoparticles (CuNPs) are actively developing and growing every year. CuNP worldwide production was estimated to be 1600 t in 2025 [1]. The global CuNP market was valued at USD 1.2 billion in 2024 and is projected to reach USD 2.5 billion by 2033 [2].

Today, CuNPs are actively used in electronics, polymer production, medicine (as antimicrobial and wound-healing agents), the textile industry as lubricating oil additives, and in the agricultural sector [3,4,5,6,7,8,9,10]. The intensive use of nanoparticles has led to increased environmental contamination through industrial and domestic wastewater discharge, leading to the pollution of water bodies, soil, and bottom sediment. Atmospheric nanoparticle emissions result in nano-sized dust deposition on various surfaces, including plant foliage, ultimately affecting all ecosystem components [11,12,13,14,15,16]. According to global estimates, 63–91% of produced engineered nanoparticles are landfilled, compared to only 0.4–7% that are released into water systems [17]. While data on copper nanoparticle release and environmental concentrations remain limited, Bakshi et al. [18] reported that about 95% of the total volume of CuNPs released into the environment ends up in soil and bottom sediments. In 2010, 36 million t of nano-sized copper entered the soil from a total production of 200 million t [19]. Copper compound toxicity generally follows this order: Cu^2+^ > nano Cu(0) > nano Cu(OH)_2_ > nano CuO > micron-scale Cu compounds [15].

Copper nanoparticles exhibit both positive and negative effects on plants depending on exposure conditions. They can improve plant nutrient utilization efficiency by employing mechanisms such as directional delivery, sustained or controlled release [20]. The potential applications of CuNPs for increasing crop yields and combating plant diseases are currently being actively investigated [21,22]. For example, synthesized CuNPs demonstrated pronounced antifungal efficacy against three fungi of the genus *Fusarium* [10]. In [23], it was shown that under drought conditions, CuNPs activate protective mechanisms in maize, thereby increasing pigment levels and antioxidant activity, total seed number, and grain yield. Malandrakis et al. [24] demonstrated that silver (AgNPs d < 100 nm), zinc oxide (ZnONPs < 50 nm), copper (CuNPs d = 25 nm), and copper oxide (CuONPs d < 50 nm) nanoparticles can inhibit the growth of seven fungal species that cause foliar and soil-borne diseases. Both AgNP and CuNP significantly suppressed *Botrytis cinerea* symptoms in vivo.

In addition to traditional root application of fertilizers and pesticides, foliar feeding is also commonly practiced. It was noted that nutrient absorption through the plant’s root system from the soil is not always effective due to leaching, soil fixation, and other losses [25]. Hong et al. [26] revealed that foliar spraying with specific amounts of micro- and macronutrients enhances the efficiency of fungicides and fertilizers, mitigating negative effects compared to traditional soil-root application. López-Vargas [27] also reported a positive result from foliar application of CuNPs. Using nanoparticle solutions at concentrations of 50, 125, 250, and 500 mg/L increased tomato fruit hardness compared to the control, thereby extending shelf life. Similarly, Nekoukhou et al. [28] observed the positive effect of CuNPs on *Dracocephalum moldavica* L. biomass, manifested by an increase in photosynthetic pigment content and essential oil yield.

At the same time, numerous studies have also described the toxic effect of CuNPs on plants, which depend on nanoparticles’ morphological parameters, physicochemical properties, solution concentrations, plant species, and environmental conditions [29,30,31]. The phytotoxicity of CuNPs stems from oxidative stress induced by ROS overproduction or copper ion release, causing cellular damage like lipid peroxidation, protein denaturation, and DNA damage [32,33]. Previous findings indicate that CuNPs can alter antioxidant enzyme activity and suppress nutrient accumulation [33]. Shi et al. [34] observed the phytotoxic effect of CuONPs on *Elsholtzia splendens* growth, primarily caused by the nanoparticles themselves. Mosa et al. [35] demonstrated that 10–30 nm CuNPs were toxic to *Crocus sativus* plants. When internalized, copper-containing nanoparticles interact with subcellular organelles and structures, inducing morphological changes. They accumulate in chloroplasts, disrupting their structure and photosynthetic activity [36]. Apodaca et al. [37] found that spraying of mint plants with copper-based compounds—including Cu(OH)_2_ nanowires, bulk Cu(OH)_2_, or ionic CuSO_4_—leads to inhibition of plant growth and nutrient accumulation. However, plant inoculation with arbuscular mycorrhizal fungi mitigated this toxicity, particularly in the root system [37]. The potential risk of CuNP accumulation in soil may result in disruption of microbial activity, highlighting the importance of evaluating CuNP interactions with plants and soil microbiota, as well as assessing trophic transfer risks. Even though CuNPs’ effect on plants or microorganisms was assessed in several studies [38,39], there is a lack of a comprehensive assessment of CuNPs’ impact on soil microbiota, plants, and human health when nanoparticles are applied through irrigation and foliar spraying in a wide range of concentrations.

The main objectives of this work are (1) to compare CuNPs translocation patterns under two exposure techniques at concentration range of 1–100 mg/L in *Mentha spicata* L., (2) to evaluate the effect of nanoparticles on *Mentha spicata* L. biochemical parameters, (3) to determine CuNPs extraction from foliage into infusion, and (4) to assess potential human health risks associated with tea consumption.

## 2. Results and Discussion

### 2.1. Nanoparticle’s Description

**Figure 1:**
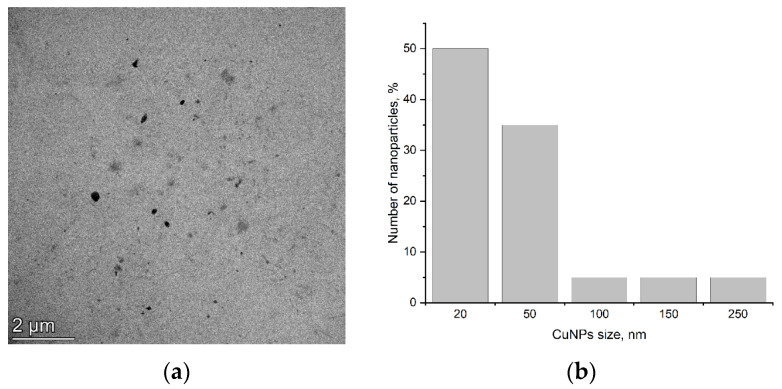
TEM image of CuNPs (**a**) and their distribution by size (**b**).

TEM images show that CuNPs are spherical in shape, with a diameter ranging from 20 to 250 nm. Most particles (85%) have a size in the range of 20–50 nm, though aggregates up to 250 nm were also observed. Figure 1 shows (a) copper nanoparticle images and (b) their size distribution.

### 2.2. Copper Uptake and Translocation in Spearmint’s Segments

The copper content in spearmint plants varies depending on the growing region. In spearmint plants collected in northwestern India, 33 elements, including copper (13.8 to 20.2 μg/g), were identified using AAS and thermal neutron activation analysis [40]. Subramanian et al. [41] reported copper levels of 29.83 ± 3.16 mg/kg in *Mentha spicata* L. Copper is a vital micronutrient for plant growth, and, generally, 5–30 mg/kg Cu is considered satisfactory in plant tissues [42].

The control soil, root, stem, and leaf samples contained 7.38, 4.77, 3.20, and 5.48 mg/kg of copper, respectively. A comparison of the copper content in soils irrigated with CuNPs (5–100 mg/L) to the maximum permissible concentration (MPC) of 40 mg/kg [43] reveals a potential 1.9- to 23.8-fold increase. This accumulation poses significant risks for the application of high-concentration copper NPs in agriculture.

**Figure 2:**
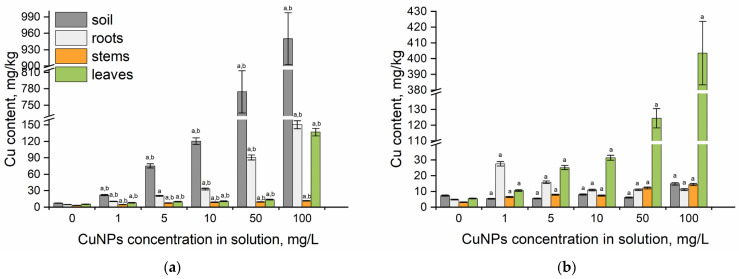
The content of copper in spearmint segments grown on soil watered with CuNPs (**a**) and exposed to foliar spraying; (**b**) a—*p* < 0.005 for the difference between experimental and control samples, and b—*p* < 0.005 for the difference between root and foliar treatments. The error bars represent the standard deviation of the measurements (n = 9).

Figure 2 presents copper content in mint segments following foliar spraying and root irrigation with CuNPs. For mint segments grown under root exposure to CuNPs, the bioconcentration and translocation factors were calculated (Table 1), while foliar-treated plants only required TF calculation.

Watering and spraying with 1–100 mg/L CuNPs caused visible changes, including leaf curling and stem curvature. The toxic effects of excess copper on plants were observed in *Mentha arvensis* L. treated with 80 mg/kg copper sulfate. The treatment caused a significant (29.2%) increase in H_2_O_2_ levels, indicating elevated ROS production, which was visually confirmed by leaf browning [44]. Metal nanoparticles can affect plants through multiple mechanisms: inducing oxidative stress and reactive oxygen species overproduction, promoting mechanical damage, for example, blockage of stomata, damaging DNA and proteins, and disrupting cellular metabolism [26]. Under root irrigation, copper was mainly accumulated in soils (maximum content 950.20 mg/kg) and roots (150.5 mg/kg), with strong positive correlations to solution concentration (soil: r = 0.96; roots: r = 0.99; *p* < 0.005). Compared to the control, the copper content in the soils increased 3–128-fold, and in the roots, it exceeded the control by 2–31 times (Figure 2a). All BCF values were <1.0, indicating limited copper uptake, probably due to NP aggregation, which reduces their availability to plants [25,26,45]. At CuNP concentrations of 1–10 mg/L, the TF for the root-stem system decreased from 0.44 to 0.08, and for the stem–leaf system from 1.7 to 1.18. However, when plants were exposed to 50 and 100 mg/L CuNPs, TFs increased from 1.45 to 11.95, respectively.

In leaves exposed to 1–50 mg/L CuNPs, copper accumulation was 1.4–2.5 times higher than in the control. At 100 mg/L CuNPs, through watering, copper content in the leaves was 137 mg/kg, which is 25 times higher than the control value (r = 0.9, *p* < 0.005). This suggests plants actively sequester potentially toxic metals in roots to limit shoot exposure [46]. Kohatsu et al. [47] revealed differences in the accumulation of CuO nanoparticles and copper salt CuSO_4_ under foliar spray versus soil irrigation. Notably, soil-applied copper (both forms) showed no leaf translocation. Similarly, copper content in soybean roots grown in soils treated with CuNPs (10–30 nm) was significantly higher than in other plant parts [48].

Foliar spraying increased copper content in stems and leaves proportionally with concentration, while showing an inverse relationship in roots (Figure 2b). The highest value of the translocation factor for the stem–root system (4.21 and 2.01, respectively) was obtained at CuNP concentrations of 1 and 5 mg/L. The highest copper uptake in roots of 27.51 mg/kg was observed at 1 mg/L of CuNPs, while at CuNP concentrations of 10–100 mg/L, it was 2.3 times higher than the control. The maximum copper uptake by leaves of 124.3 and 403.5 mg/kg, exceeding the control values by 22 and 73 times, respectively, was found to be attained at CuNP concentrations of 50 mg/L and 100 mg/L (Pearson’s correlation coefficient r = 0.98 at *p* < 0.005). These results align with Xiong et al. [49], where CuO nanoparticles were foliar-applied; CuO nanoparticles primarily accumulated in lettuce and cabbage leaves (0–250 mg/plant) [49]. Sprayed nanoparticles primarily enter the leaves through stomata and are transported in plant tissues; however, leaf wax and cell walls can create barriers to their absorption. It was reported that some nanoparticles were deposited in the form of micrometric aggregates either on the surface of leaves or in stomata [26,49,50]. Keller et al. [45] identified species-dependent accumulation of CuO nanoparticles influenced by leaf surface hydrophilicity and cuticle roughness. The highest amount was found on the surface of lettuce leaves (*Lactuca sativa*), followed by collard green cabbage (*Brassica oleracea*, var. *Acephala*), and then kale (*Brassica oleracea*, var. *Acephala Lacinato*) [45].

The significant differences in copper accumulation between the foliar and root pathways are mainly due to the combined effect of soil parameters and the root barrier’s function, which can substantially limit metal bioavailability to the plant during root uptake [51]. Besides immobilization in the soil, a key factor determining the bioavailability of CuNPs during root uptake is their chemical transformation in the rhizosphere [52]. However, physicochemical interactions between NPs and root cells can affect the integrity and structure of the cell wall, causing the expansion of pores, which, in turn, facilitates NP penetration in roots [48]. Cuticular and stomatal uptake are two potential pathways for nanoparticle entry into the plant leaves [53]. Higher uptake of CuNPs in leaves in this case can be explained by a higher concentration gradient, as water evaporates from the leaf surface, leaving the NPs to be absorbed by the leaf [54].

### 2.3. Effect of Foliar Application and Irrigation of CuNPs on Spearmint Biochemical Parameters

**Figure 3:**
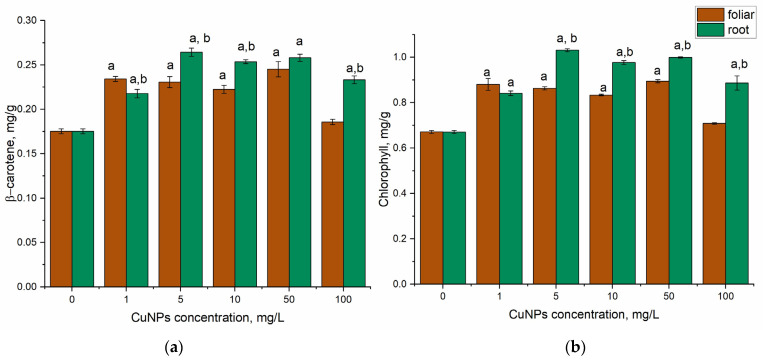
Change in the content of (**a**) β-carotene and (**b**) chlorophyll under the action of CuNPs (root and foliar treatment: a—*p* < 0.05 for the difference between experimental and control samples; b—*p* < 0.05 for the difference between root and foliar treatments. The error bars represent the standard deviation of the measurements (n = 9).

Mint plant irrigation with CuNPs stimulated an increase in β-carotene content in plants at all applied concentrations (Figure 3a). The increase in pigment content in leaves varied from 24% at CuNPs concentration of 1 mg/L to 51% (*p* < 0.05) at NPs concentration of 5 mg/L. At a CuNP concentration of 10–100 mg/L, the increase in β-carotene content was at the level of 33% (*p* < 0.05)—47% (*p* < 0.05) compared to the control. The same pattern was observed in plants sprayed with CuNPs. The level of β-carotene in leaves increased by 6–40% and the most pronounced increase was observed at CuNPs concentration of 50 mg/L.

In mint leaves treated with CuNPs by spraying, the content of chlorophyll increased by 24 (*p* < 0.05)—33% compared to the control at NPs concentration range of 1–50 mg/L (Figure 3b). A CuNP concentration of 100 mg/L did not significantly modify the pigment content of the leaves. Watering plants with CuNPs had a greater impact on chlorophyll content. Thus, at CuNP concentrations of 1 and 100 mg/L, the increase was 25% and 32%, respectively. Application of CuNPs in concentrations of 5–50 mg/L stimulated an increase in chlorophyll content in leaves by 50% (*p* < 0.05) compared to control samples. Such a response of plants to CuNPs can be explained by their properties, which stimulate the presence of enzymatic antioxidants that scavenge free radicals, protecting plants from ROS and consequently from disruption of cellular function [55].

**Figure 4:**
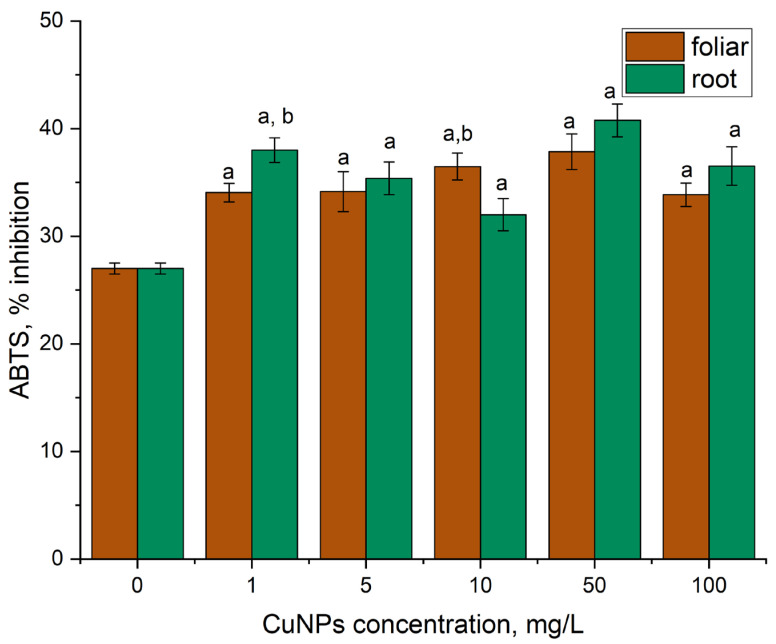
Modification of the antioxidant activity of ethanolic extracts obtained from mint leaves under the action of CuNPs (root and foliar treatment): a—*p* < 0.05 for the difference between experimental and control samples; b—*p* < 0.05 for the difference between root and foliar treatments. The error bars represent the standard deviation of the measurements (n = 9).

Treatment of mint plants with CuNPs led to an increase in antioxidant activity in the case of both routes of exposure (Figure 4). Spraying of mint plants with CuNPs in a concentration range of 5–50 mg/L led to an increase in antioxidant activity by 26–40% compared to the control. The increase in the level of the antioxidant activity at the application of the highest CuNP concentration was comparable to the effect seen at CuNP concentrations of 1 and 5 mg/L, the increase being of 25%. Plant irrigation with NPs showed a more pronounced increase in the antioxidant activity values, by 18–51% (*p* < 0.05), compared to the control.

Raza et al. [56] showed that the application of CuNPs significantly affects growth and yield under both normal and drought conditions. Application of CuNPs can effectively alleviate drought stress, leading to improved growth and yield of wheat, especially under controlled conditions [56]. The positive effect of foliar NP treatment on biochemical contents was noted for *Capsicum annuum* L. plants, which was manifested in a significant increase in the content of chlorophyll and carotenoids [57]. Application of CuNPs as nanofertilizers led to a pronounced increase in the antioxidant activity of French basil seedlings (*Ocimum basilicum* L var. Grand Vert) and stimulated photosynthetic pigments [58]. However, the root treatment of parsley with CuNPs negatively affected the amount of pigments and antioxidant activity [38]. The chlorophyll content in fresh tomato foliage was reduced by 32–70% upon application of CuONPs [36].

### 2.4. Soil Microbial Activity

**Figure 5:**
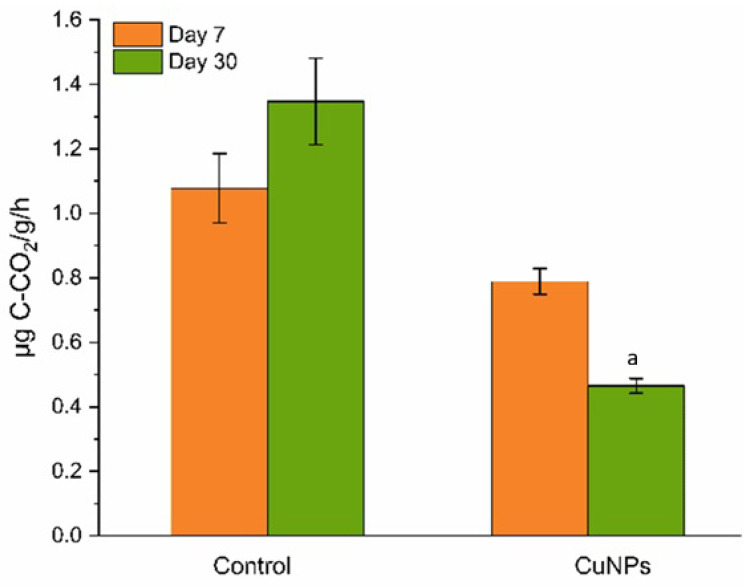
Soil respiration rates in root irrigation treatments with copper nanoparticles (CuNPs) at concentrations of 0 mg/L (Control) and 100 mg/L (CuNPs), measured after 7 and 30 days of incubation: a—*p* < 0.05 for the difference between experimental samples after 7 and 30 days of incubation.

Root irrigation with CuNPs at a concentration of 100 mg/L resulted in a statistically significant inhibition of soil microbial activity. Throughout the 30-day incubation period, soil respiration in the CuNP-treated variant was consistently lower than in the control and exhibited a decreasing trend over time (Figure 5). Specifically, respiration in the CuNP-treated soil was 27% and 65% lower than the control on days 7 and 30, respectively, representing a three-fold reduction by the end. Between these two time points, respiration in the CuNP-treated samples decreased by 41%, whereas in the control, it increased by 25%.

The observed inhibitory effect of CuNPs differed from those reported in our previous studies using other plants and/or nanoparticles under similar experimental conditions. Notably, when calendula (*Calendula officinalis* L.) was irrigated with CuNPs, the inhibition was milder—only 38% compared to the control [39]. Similarly, when gold nanoparticles (AuNPs) were used instead of CuNPs, the inhibition was 28% and the most pronounced inhibition—73%—was observed in the case of calendula irrigated with silver nanoparticles (AgNPs) [39,59].

These findings suggest that CuNPs at concentrations of 100 mg/L can exert a considerable toxic effect on soil microbial activity. Their impact is comparable to that of AgNPs, which are widely recognized for their strong biocidal properties [60]. However, the degree of toxicity appears to vary depending on the time elapsed after application and the plant species involved. Overall, CuNPs pose a significant environmental risk, and further research is needed to establish guidelines for their safe use in soil systems.

### 2.5. Assessment of Copper Transfer from Plants in the Infusion and Health Risk Assessment

Due to the active use of spearmint for preparing herbal infusions, copper extraction from leaves during brewing was assessed. Copper extraction in solution ranged from 3 to 55% for both types of exposure and constituted 64% for the control (Table 2).

The copper concentration in the infusions obtained from mint treated with CuNPs through root irrigation was in the range of 0.125–0.215 mg/L. A weak correlation was observed between the copper concentration in the herbal infusion and its content in dried leaves (r = 0.79 at *p* < 0.005). The copper concentration in infusions prepared from nanoparticle-sprayed leaves ranged from 0.238 mg/L to 1.53 mg/L. In this case, the copper concentration in infusion was directly proportional to the content in leaves (r = 0.97 at *p* < 0.005). The percentage of copper extraction from mint leaves is comparable to the data obtained for mixed herbal infusions from North Ossetia (Caucasus), where extraction values were in the range from 16 to 57% [61].

The EDI values for herbal tea prepared from leaves sprayed with CuNPs were 1.4–3.7 times higher compared to plants watered with CuNPs (Table 3). EDI increased with increasing copper concentration in herbal infusion (when mint plants were sprayed with NPs), reaching a value of 1.53 mg/L. In the case of root exposure, copper extraction into the infusion ranged from 0.125 to 0.215 mg/L, and the EDI values differed slightly from the control. HQ values were <1 in all experimental variants, which allows for the conclusion that there are no health risks from long-term consumption of copper-containing mint infusion with a Cu concentration not exceeding 1.53 mg/L.

## 3. Materials and Methods

### 3.1. Materials

Spearmint (*Mentha spicata* L.), a perennial herbaceous plant of the genus *Mentha*, family *Lamiaceae*, was used as the object of study. Spearmint is successfully cultivated in many countries of the world, commonly grown in home gardens [62]. The aerial parts of spearmint are used for essential oils production, and as flavoring in beverages, confectioneries, chewing gum, ice cream, salads, soups, desserts, etc. [63,64,65].

For experiments, universal nutrient soil C-2 (VELTORF LLC, Velikie Luki, Russia), a peat-based mixture containing grade 1:1:1 nitroammophoska from nitric acid decomposition was used. The initial solution of CuNPs of chemical purity (coated with polyvinylpyrrolidone and stable for two months), produced by the M9 company (Tolyatti, Russia), had a concentration of 200 mg/L. For the experiments, the concentrate was diluted with deionized water to obtain working solutions in concentrations of 1, 5, 10, 50, and 100 mg/L. For each plant treatment, fresh working solutions were prepared.

### 3.2. Mentha Treatment with CuNPs

*Mentha spicata* L. root cuttings were planted in 200 mL pots filled with soil. Nanoparticle exposure began two weeks after shoot emergence. Plant irrigation and foliar spraying were performed once every 2 days, using CuNP solutions (1–100 mg/L). Root application is a classic agronomic method of fertilizer application, while foliar application is used to quickly correct nutrient deficiencies, especially micronutrients in plants [66]. The volume of solution applied during one-time irrigation of plants was 20 mL, fully satisfying the plant’s moisture needs. Foliar spraying was performed using a polypropylene aerosol. To prevent solutions running off from the leaves, an optimal volume of 2 mL of solution was applied to the surface of one plant foliage at a time. The penetration of nanoparticle solutions into the soil was prevented by its screening. The experiment lasted for 28 days, after which soil and plants, divided into roots, as well as stems, and leaves, were dried at 50 °C for 72 h, homogenized, and then dried at 105 °C for 24 h. For biochemical analysis, fresh leaves were collected and frozen at −80 °C. Experiments were performed in three repetitions.

### 3.3. Herbal Tea Preparation

To prepare infusions, 0.5 g of crushed dried mint leaves was poured with 10 mL of deionized water, heated to 80 °C. The resulting herbal decoctions were brewed under closed lids for 5 min, and after that, ash-free filters were used for filtration. The temperature and duration of brewing were selected in accordance with the recommendations of manufacturers of commercial herbal products (dried crushed mint leaves). According to [67], the highest solubility of many elements, including copper, occurs during the first 5 min of brewing [67].

### 3.4. Analytical Techniques

Transmission electron microscope images were obtained by using Thermo Scientific Talos F200i (Thermo Scientific, Waltham, MA, USA), which allowed determining the size and shape of nanoparticles. The copper content in soil and plant samples was determined by the PlasmaQuant PQ 9000 Elite high-resolution optical emission spectrometer with inductively coupled plasma (Analytik Jena, Jena, Germany). Sample preparation was performed according to the procedure described by [41]. Quality control of measurements was ensured by analysis of certified reference materials INCT PVTL-6 (Polish Virginia Tobacco Leaves), NIST 1573a (Tomato Leaves), and NIST 2709a (San Joaquin Soil Baseline Trace Element Concentrations). The error of determination was less than 5%. Each sample was measured in three repetitions.

### 3.5. Biochemical Analysis and Antioxidant Activity

#### 3.5.1. Pigments Extraction and Quantification (Mint Leaves)

Pigments from fresh mint leaves biomass were extracted using acetone (90%, *v*/*v*) at a ratio of 10 mg fresh biomass per 1.0 mL solvent. Samples were vortexed and kept under gentle shaking for 120 min at room temperature; the extracts were separated from the biomass by centrifugation. Absorbance of the supernatant was recorded in 1 cm cuvettes on a T60 Visible Spectrophotometer (PG Instruments Ltd., Lutterworth, UK) using 90% acetone as the blank. For chlorophyll determination, spectra were read at 663 nm and 645 nm; for total carotenoids, at 470 nm. Concentrations were calculated with the Lichtenthaler equations [68] (acetone system), and total chlorophyll is presented as the sum of Chl a + Chl b. Results were expressed as mg/g fresh weight (FW).

#### 3.5.2. Antioxidant Activity

Spearmint extract was prepared using a 50% aqueous ethanol solution. Antioxidant activity was assessed using the ABTS assay according to the spectrophotometric method [69], which relies on the decolorization of the ABTS radical cation. The radical was generated by reacting 7 mM ABTS with 2.45 mM potassium persulfate (1:1, *v*/*v*) in the dark for 16 h, and subsequently diluted with 50% ethanol to reach an absorbance of 0.70 ± 0.02 at 750 nm.

The reaction mixture was prepared by mixing 2.7 mL of the ABTS solution with 0.3 mL of the extract. The reaction was allowed to proceed for 6 min, a standardized and widely accepted reaction time that enables the comparison of results across different laboratories. This reaction time is also accepted for a wide range of plant extracts, which typically involve the extraction of complex antioxidant compounds. The standardized timing makes the method universal for monitoring the evolution of antioxidant activity, regardless of the plant species, plant part, or extraction system employed [70]. Subsequently, the absorbance was measured, and the percentage of ABTS radical inhibition was calculated.

### 3.6. Soil Microbial Activity Measurment

Soil microbial activity, measured as the soil respiration rate, was assessed following the method described in [59]. Briefly, the measurement involved monitoring the increase in CO_2_ concentration within a closed dynamic chamber. The system comprised an Erlenmeyer flask containing a soil sample, connected via tubing to an infrared gas analyzer Li-850 (LI-COR).

Two plant treatment variants were analyzed: a control (no CuNPs) and a treatment involving root irrigation with CuNPs at a concentration of 100 mg/L. Soil samples were collected at the conclusion of the plant experiment and stored in an air-dried state for approximately one month. Prior to analysis, 25 g samples of each soil (in triplicate) were placed into 250 mL Erlenmeyer flasks, remoistened with 9 mL of distilled water, and incubated in the dark at 25 °C for 30 days. Moisture levels were periodically adjusted throughout the incubation. Soil respiration rates were measured on days 7 and 30.

### 3.7. Data Evaluation

The content of copper in plant segments and soil was used to calculate the bioconcentration factor and translocation coefficients [71]. The bioconcentration factor (*BCF*) (1) was calculated in the case of root irrigation and was defined as the ratio of the content of copper in plant segments to its content in soil (*C_S_*) and the translocation factor (*TF*) was calculated as the ratio of copper content in stems to its content in roots (*C_R_)*, in leaves (*C_L_*) to its content stems (*C_St_*), and in leaves to its content in roots (2). The calculation was analogous to that described in [59].

(1) $$BCF=\frac{C_{R\;(St,\;\;\;L)}}{C_{S\;}}$$

(2) $$TF=\frac{C_{St\;or\;L}\;}{C_{R\;or\;St}}$$

When the leaf surface was exposed to nanoparticle solutions, the translocation of copper from the aerial part to the root system was calculated as the ratio (3):

(3) $$TF=\;\frac{C_{R\;or\;St}\;}{C_{St\;or\;L}}$$

The estimated daily intake (*EDI*) of copper was calculated according to Equation (4):

(4) $$EDI=\frac{C_{i}*IR*EF*ED}{AT*BW}$$

where *C_i_*—copper concentration in the tea infusion (mg/L);

*IR*—Tea ingestion rate (1250 mL per person per day);

*EF*—Exposure frequency (365 days/year);

*ED*—Exposure duration (70 years);

*AT*—Averaging time, calculated as (*EF* × *ED*);

*BW*—Average body weight (70 kg).

The hazard quotient (*HQ*) of copper in tea infusion was calculated to qualitatively assess the non-carcinogenic effects of metal on humans through tea consumption (5):

(5) $$HQ=\frac{EDI}{RfD}$$

The potential non-carcinogenic risk from copper in herbal infusions was assessed by calculating the estimated daily intake (EDI) and hazard quotient (*HQ*). The calculations were performed using established equations, where the oral reference dose (*RfD*) is the daily oral reference dose for copper 0.04 mg·kg/day [72]. For detailed parameters and equations, see the Human Health Risk Assessment section [59].

### 3.8. Statistical Analysis

Statistical analysis was performed with one-way analysis of variance (ANOVA) by the use of Statistica 10 (Student’s *t*-tests). The results are presented as average ± standard deviation.

## 4. Conclusions

The uptake of copper nanoparticles by mint plants exposed to CuNPs through two application methods was investigated during a 28-day experiment. Foliar exposure resulted in greater copper accumulation in the aerial parts of mint compared with root irrigation, which primarily led to copper accumulation in soil and roots. When irrigated with 1–100 mg/L CuNPs, copper content in plant segments changed in the following order: soil > roots > leaves > stems. Soil respiration rates at copper concentrations of 950.2 mg/kg revealed substantial toxicity of CuNPs to microbial activity, highlighting a potential environmental risk associated with their accumulation in soil. Further investigation is required to assess the long-term and dose-dependent ecotoxicological impacts of CuNPs on soil systems.

Foliar spraying experiments demonstrated mint’s ability to translocate copper from shoots to roots, particularly at CuNPs concentrations of 1–5 mg/L. At 1 mg/L, the maximum copper content was observed in roots (27.51 mg/kg), followed by the following: leaves > stems > soil. However, higher concentrations reduced root copper levels, likely due to stomatal blockage by nanoparticles. Spraying of plants with CuNPs in the concentration range of 10–100 mg/L caused an increase of copper content in leaves by 5.7–73.6 times compared to the control. Despite these effects, both application methods stimulated pigment production, increased chlorophyll and carotenoid content, and enhanced antioxidant activity.

Tea brewing experiments revealed a direct proportionality between copper concentration in the infusion and its content in the leaves for foliar-treated plants. A weaker correlation was observed for root-irrigated plants. The EDI and HQ values indicate no health risks from consuming infusions containing 0.125–1.53 mg/L of copper.

*Study limitations and further research*: A comprehensive assessment of the ecotoxicological effects of CuNPs on soil microbiota requires further study. This research should investigate a wider range of NP concentrations and account for their physicochemical properties, such as size, shape, and surface characteristics. Since increases in antioxidant activity and level of pigments may indicate both a stimulating (hormetic) effect or the response to oxidative stress, this phenomenon needs a more detailed investigation. The bioavailability of CuNPs varies greatly depending on their physicochemical properties, the plant species, and environmental conditions; further studies are necessary. These should assess the impact of these parameters on NP uptake, preferably in long-term experiments.

## Figures and Tables

**Table 1:** Bioconcentration factors (BCFs) and translocation factors (TFs) in spearmint segments under root irrigation and foliar spraying treatment with CuNPs.

Concentration of CuNPs in Solution, mg/L	BCF			TF		
	Roots	Stems	Leaves	Stems/Roots	Leaves/Stems	Leaves/Roots
Root irrigation						
1	0.48 ± 0.014	0.21 ± 0.006	0.36 ± 0.01	0.44 ± 0.01	1.70 ± 0.05	1.1 ± 0.01
5	0.27 ± 0.008	0.10 ± 0.003	0.13 ± 0.004	0.37 ± 0.01	1.31 ± 0.03	0.8 ± 0.003
10	0.28 ± 0.008	0.08 ± 0.002	0.09 ± 0.003	0.28 ± 0.008	1.18 ± 0.03	0.5 ± 0.004
50	0.12 ± 0.003	0.01 ± 0.0003	0.02 ± 0.0006	0.11 ± 0.003	1.45 ± 0.04	0.3 ± 0.008
100	0.16 ± 0.005	0.01 ± 0.0003	0.14 ± 0.004	0.08 ± 0.0001	11.95 ± 0.35	0.2 ± 0.001
Foliar spraying						
				Roots/Stems	Stems/Leaves	Roots/Leaves
1				4.21 ± 0.13	0.62 ± 0.01	2.62 ± 0.08
5				2.01 ± 0.06	0.31 ± 0.009	0.63 ± 0.02
10				1.49 ± 0.04	0.23 ± 0.007	0.35 ± 0.01
50				0.91 ± 0.027	0.10 ± 0.003	0.09 ± 0.003
100				0.77 ± 0.023	0.04 ± 0.001	0.03 ± 0.0009

**Table 2:** Copper extraction from leaves into infusion.

	Copper Content in Leaves Under Root Exposure, mg/kg	Extraction, %	Copper Content in Leaves Under Foliar Spraying Condition, mg/kg	Extraction, %
Control	5.48 ± 0.16	64	5.48 ± 0.16	64
1	8.03 ± 0.24	31	10.5 ± 0.3	55
5	9.92 ± 0.3	33	25.1 ± 0.7	24
10	10.81 ± 0.3	31	31.4 ± 0.9	37
50	13.83 ± 0.4	23	124.3 ± 3.7	10
100	137 ± 4.1	3	403.5 ± 12.1	8

**Table 3:** Estimated daily intakes (EDIs) and hazard quotient (HQ) of copper due to the herbal tea consumption brewed from the spearmint leaves.

	Copper Content in Infusion, mg/L		EDI		HQ	
	Root Exposure	Foliar Spraying Condition	Root Exposure	Foliar Spraying Condition	Root Exposure	Foliar Spraying Condition
Control	0.175 ± 0.05	0.175 ± 0.05	3.13 × 10^−3^	3.13 × 10^−3^	7.81 × 10^−2^	7.81 × 10^−2^
1	0.125 ± 0.004	0.238 ± 0.007	2.23 × 10^−3^	3.13 × 10^−3^	5.56 × 10^−2^	1.1 × 10^−1^
5	0.165 ± 0.005	0.242 ± 0.007	2.95 × 10^−3^	4.25 × 10^−3^	7.38 × 10^−2^	1.1 × 10^−1^
10	0.169 ± 0.05	0.583 ± 0.01	3.01 × 10^−3^	4.33 × 10^−3^	7.53 × 10^−2^	2.6 × 10^−1^
50	0.158 ± 0.004	0.619 ± 0.02	2.83 × 10^−3^	1.04 × 10^−2^	7.07 × 10^−2^	2.8 × 10^−1^
100	0.215 ± 0.006	1.53 ± 0.04	3.85 × 10^−3^	1.11 × 10^−2^	9.62 × 10^−2^	6.8 × 10^−1^

## Data Availability

Data are contained within the article.
